# A new neuroanatomical two-dimensional fitting three-dimensional imaging techniques in neuroanatomy education

**DOI:** 10.1186/s12909-023-04323-z

**Published:** 2023-05-14

**Authors:** Xuefei Shao, Di Qiang, Quan Yuan

**Affiliations:** 1 Department of Neurosurgery, The First Affiliated Hospital of Wannan Medical Collgeg (Yijishan Hospital of Wannan Medical Collgeg), Wuhu, China; 2 Department of Dermatology and STD, The First Affiliated Hospital of Wannan Medical Collgeg (Yijishan Hospital of Wannan Medical Collgeg), Wuhu, China; 3 Department of Imaging, The First Affiliated Hospital of Wannan Medical Collgeg (Yijishan Hospital of Wannan Medical Collgeg), No.22 Road, Wuhu city, 241002 Anhui Province China

**Keywords:** Virtual reality, Neuroanatomic education, Two-dimensional three-dimensional techniques, Hand-held scanner 3D imaging techniques

## Abstract

**Background:**

Neuroanatomy is the most abstract and complex anatomy. Neurosurgeons have to spend plenty of time mastering the nuances of the autopsy. However, the laboratory that can meet the requirements of neurosurgery microanatomy is only owned by several large medical colleges because it is an expensive affair. Thus, laboratories worldwide are searching for substitutes,but the reality and local details might not meet the exact requirements of the anatomical structure. Herein, we compared the traditional teaching mode, the 3D image generated by the current advanced hand-held scanner and our self-developed 2D image fitting 3D imaging method in the comparative study of neuroanatomy education.

**Methods:**

To examine the efficacy of two-dimensional fitting three-dimensional imaging techniques in neuroanatomy education. 60 clinical students of grade 2020 in Wannan Medical College were randomly divided into traditional teaching group, hand held scanner 3D imaging group and 2D fitting 3D method group, with 20 students in each group.First, the modeling images of the hand held scanner 3D imaging group and the 2D fitting 3D method group are analyzed and compared, and then the teaching results of the three groups are evaluated by objective and subjective evaluation methods. The objective evaluation is in the form of examination papers, unified proposition and unified score; The subjective evaluation is conducted in the form of questionnaires to evaluate.

**Results:**

The modeling and image analysis of the current advanced hand-held 3D imaging scanner and our self-developed 2D fitting 3D imaging method were compared.The images (equivalent to 1, 10, and 40 × magnification) of the model points and polygons using the Cinema 4D R19 virtual camera of 50, 500, and 2000 mm showed 1,249,955 points and 2,500,122 polygons in the skull data obtained using the hand-held scanner. The 3D model data of the skull consisted of 499,914 points, while the number of polygons reached up to 60,000,000, which was about fourfold that of the hand-held 3D scanning. This model used 8 K mapping technology, and hand-held scanner 3D imaging 3D scanning modeling used a 0.13 K map based on the map data, thereby indicating that the 2D fitting 3D imaging method is delicate and real. Comparative analysis of general data of three groups of students.The comparison of test results, clinical practice assessment and teaching satisfaction of the three groups shows that the performance of hand held scanner 3D imaging group is better than that of traditional teaching group (*P* < 0.01), and that of 2D fitting 3D method group is significantly better than that of traditional teaching group (*P* < 0.01).

**Conclusions:**

The method used in this study can achieve real reduction. Compared to hand-held scanning, this method is more cost-effective than the cost of the equipment and the results. Moreover, the post-processing is easy to master, and the autopsy can be performed easily after learning, negating the need to seek professional help. It has a wide application prospect in teaching.

**Supplementary Information:**

The online version contains supplementary material available at 10.1186/s12909-023-04323-z.

## Background

Anatomy is an essential introductory course in medical study hat that must be mastered. Cadaver specimen is the most direct and main tool of anatomic learning, and the contradiction between the increasing demand and the shortage of specimen sources is becoming prominent [1].

Supposedly, the construction of students’ sense of space has always been a challenge in anatomy teaching. With multimedia and computer digital operation platform, a large amount of real human cross-section data information is integrated into the computer to reconstruct the three-dimensional (3D) structural image of the human body, i.e., the use of the digital virtual human body. It provides many auxiliary teaching tools of sectional anatomy, systematic dissection, and regional anatomy for medical teaching, which is the combination of medicine, information technology, and computer technology. Combined with the results, the teaching path of anatomy has been innovated but is still far from the requirements of various medical specialties because it is difficult to realize the true reduction [2, 3].

Neuroanatomy is the most abstract and complex anatomy. Neurosurgeons have to spend plenty of time mastering the nuances of the autopsy. However, the laboratory that can meet the requirements of neurosurgery microanatomy is only owned by several large medical colleges because it is an expensive affair. Thus, laboratories worldwide are searching for substitutes, leading to the boom of 3D scanning and printing. Although significant progress has been made, the reality and local details might not meet the exact requirements of the anatomical structure. Therefore, efforts are been made to achieve realistic images by creating and rendering 3D models. However, the process is tedious and multidisciplinary cooperation is needed [4]. At present, neuroanatomy still uses red and blue glasses to see the stereoscopic effect of the anatomical pictures through the principle of parallax of eyes, but the effect is limited. In the present study, a new imaging method is applied to neuroanatomy. Herein, we compared the traditional teaching mode, the 3D image generated by the current advanced hand-held scanner and our self-developed 2D image fitting 3D imaging method in the comparative study of neuroanatomy.

## Methods

### Student information and teaching methods

Sixty clinical students were randomly divided into traditional teaching group, hand held scanner 3D imaging group and 2D fitting 3D method group, with 20 students in each group.

**Fig. 1:**
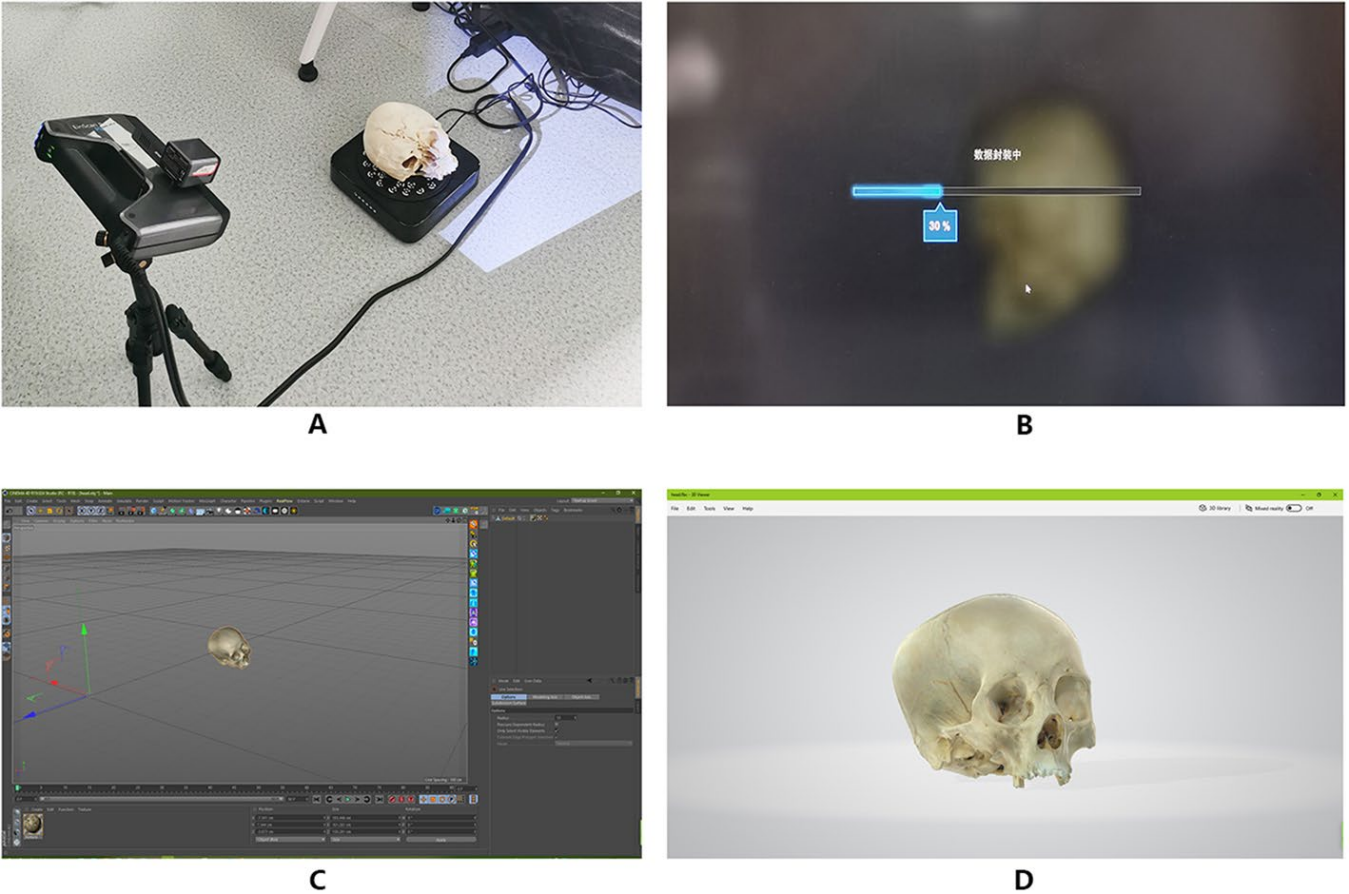
The step of scanning scene of skull specimen with hand-held scanner
- 3.The teaching method based on 2D fitting 3D method group modeling is as follows.
- Equipment: Canon EOS r camera (model: EOS r manufacturer: Canon Inc.), Canon ef100 mm macro (model: ef100 mm macro manufacturer: Canon Inc.), Manfrotto professional tripod (model: Manfrotto mk190xpro4-3w PTZ model: mhx-pro-3w manufacturer: Vitec imaging solutions SPA), professional step turntable, black background cloth and bracket, large ring continuous light source.
  - 3.1 2D shooting method and steps (the skull is used as an example)
  - 3.1.1 The black background cloth with a special bracket was arranged, the professional stepping turntable was placed on it, and the skull specimen was placed on the turntable to ensure that the axis of the specimen and the turntable are consistent. A circular continuous light source was placed to illuminate the skull specimen evenly. The camera was fixed on a tripod that was adjusted to a horizontal position. The focus of the camera was on the skull specimen, and the camera was set up in the M position (Fig. 2A.B).

- 1.The traditional teaching group uses the traditional teaching method to systematically tell the basic theoretical knowledge and auxiliary multimedia, and systematically teach chapter by chapter according to the requirements of the teaching syllabus.
- 2.The teaching method of the hand held scanner 3D imaging group is based on the hand held scanner 3D imaging modeling. The details are as follows.
  Equipment: The source of the cadaver specimen from the Anatomy Laboratory of the Wannan Medical College. For dissection, each cadaveric head was injected with colored silicone; the arterial system was injected with red silicone and blue for the venous system.The study was approved by the institutional review board of Wannan Medical College. Einstein Pro 2 × plus2020 (model: Pro 2 × plus2020, manufacturer: Shipping 3D Technology Inc.), small tripod, ordinary turntable (the skull is used as an example). First, calibrate the hand-held scanner. The skull specimen was placed on the turntable, and the specimen was placed vertically. The scanner was supported by a small tripod. The specimen was scanned at the same time. After the turntable was rotated 720°, the specimen was placed horizontally and scanned to supplement the scanning position (Fig. 1A). Subsequently, the white model is generated, and then the map and package are generated (Fig. 1B).

**Fig. 2:**
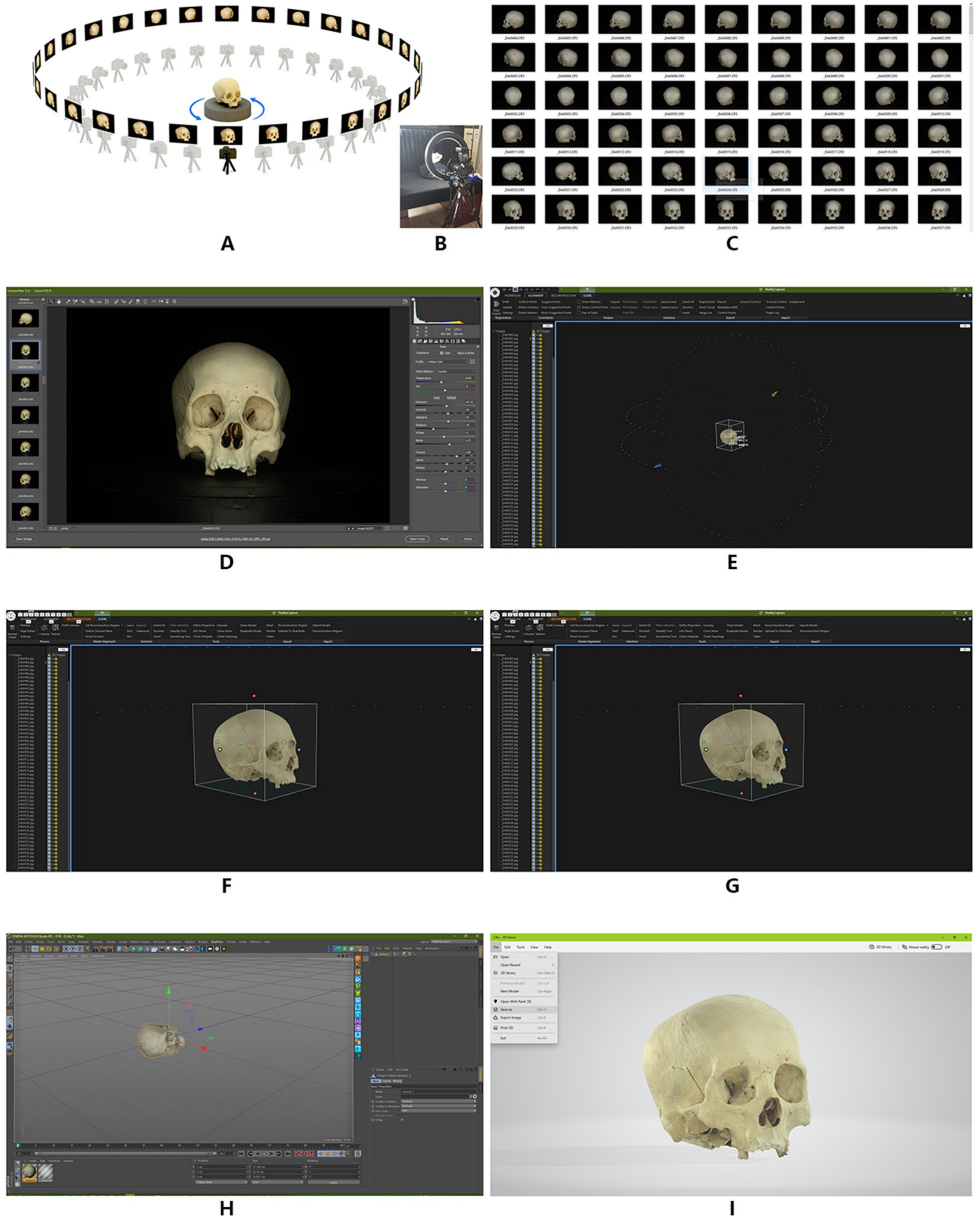
The step of scanning scene of skull specimen with two-dimensional fitting three-dimensional imaging techniques
  - 3.1.2 The turntable was set at 1/64 step, and the specimens were placed vertically at 1/64 × 360°, i.e., 5.625°. A photo taken of the specimen is about 30.3 million pixels, and the CR3 format file is generated. After 360° rotation, 64 CR3 format files were obtained. Then, the specimens were placed horizontally, and the above steps were repeated. Finally, some parts that could not be photographed or blocked by normal view angles were photographed, obtaining a total of 207 CR3 format files (Fig. 2C).
  - 3.1.3 The CR3 format file was imported into Adobe Photoshop cc2015 and processed with a camera raw 12.0 to retain the details of the highlight and shadow parts of the specimen that is developed into a JPEG image file (Fig. 2D).
- 3.2 Methods and steps of 3D model reconstruction
  - 3.2.1 The JPEG format file sequence is imported into the software reality capture beta 1.0 at a time. After image alignment, control points are added for 3D model reconstruction and texture mapping (Fig. 2E. F).
  - 3.2.2 After completion, the obj format model file, which contains MTL material file and PNG texture file is imported into Cinema 4D R19, and exported to FBX format file after resetting the object coordinates; also, the file volume is compressed (Fig. 2G. H).
  - 3.2.3 FBX format file is opened by Windows 10 3D viewer software and saved as a GLB format file to further compress the file volume (Fig. 2I).
    - 1.2 After completion, the obj format model file containing MTL material file and PNG texture file is imported into Cinema 4D R19. After the object coordinates are reset, the file is exported to FBX format to compress the file volume (Fig. 1C).
    - 1.3 FBX format file is opened by Windows 10 3D viewer software and saved as a GLB format file to further compress the file volume (Fig. 1D).

### Teaching effectiveness evaluation

First, the modeling images of the hand held scanner 3D imaging group and the 2D fitting 3D method group are analyzed and compared, and then the teaching results of the three groups are evaluated by objective and subjective evaluation methods. The objective evaluation is in the form of examination papers, unified proposition and unified score; The subjective evaluation is conducted in the form of questionnaires to evaluate.

### Statistical analysis

The SPSS18.0 statistical software program was used for data analysis. The teaching effect of the three groups was compared. The statistical description of categorical variables was number of cases (percentage), and the statistical inference was chi square test. The mean ± standard deviation is used when the data of continuous variables meet the normal distribution, and the median (quartile) is used when the data do not meet the normal distribution; One way ANOVA or rank sum test was used for statistical inference, and LSD method or nemenyi method was used for pairwise comparison. *P* < 0.05 was statistically significant.

## Results

### Comparison of modeling results

**Fig. 3:**
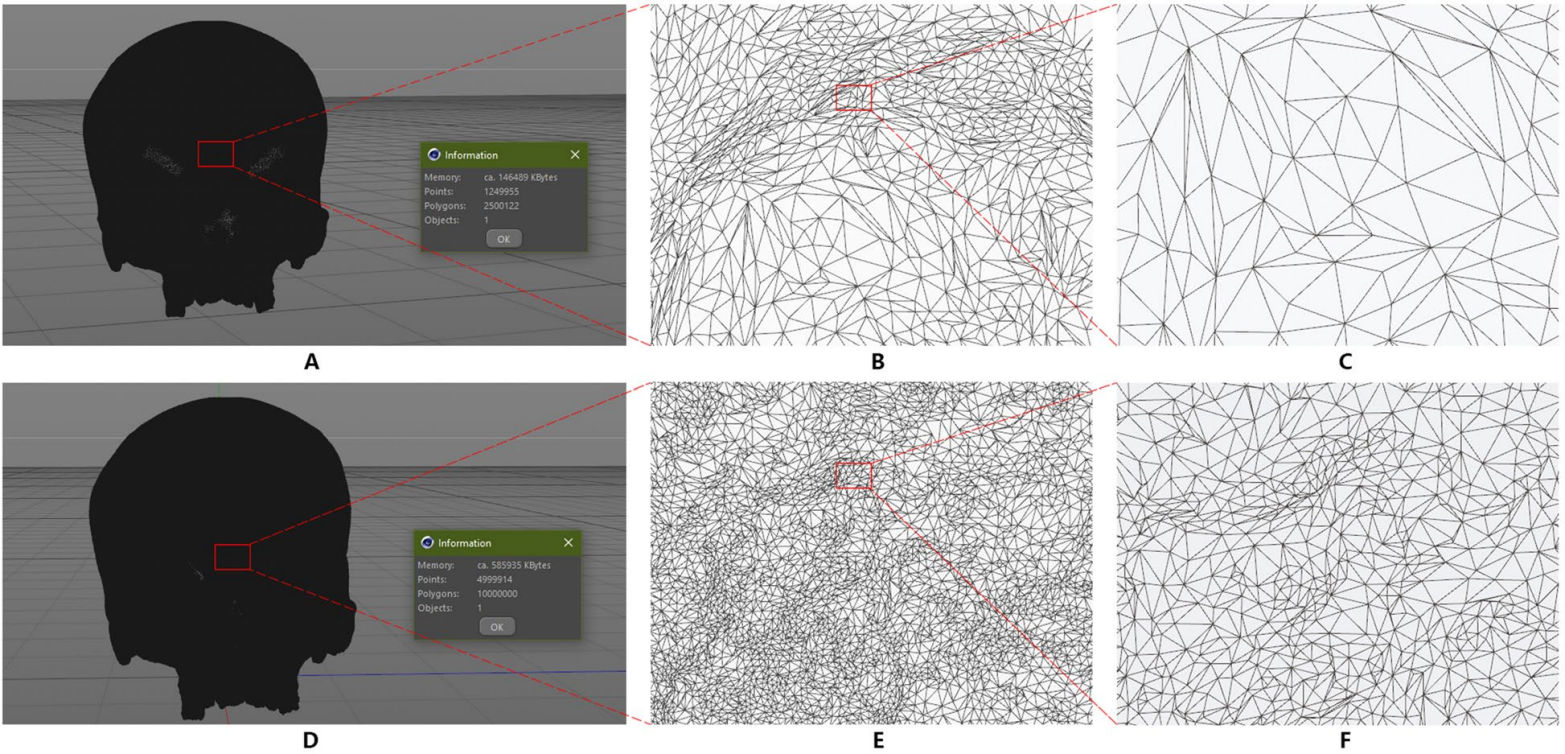
Comparison of two methods to generate model data

The data analysis of the images (equivalent to 1, 10, and 40 × magnification) of the model points and polygons using the Cinema 4D R19 virtual camera of 50, 500, and 2000 mm showed that there were 1,249,955 points and 2,500,122 polygons in the skull data of the hand-held scanner. The 3D model data of the skull consisted of 499,914 points. The number of polygons can reach up to 60,000,000, which is about fourfold of hand-held 3D scanning modeling. This research model uses 8 K mapping technology based on the map data, and hand-held scanner 3D imaging 3D scanning modeling uses 0.13 K mapping (Fig. 3).

### Details of the same location of the skull (Figs. 4 and 5)

**Fig. 4:**
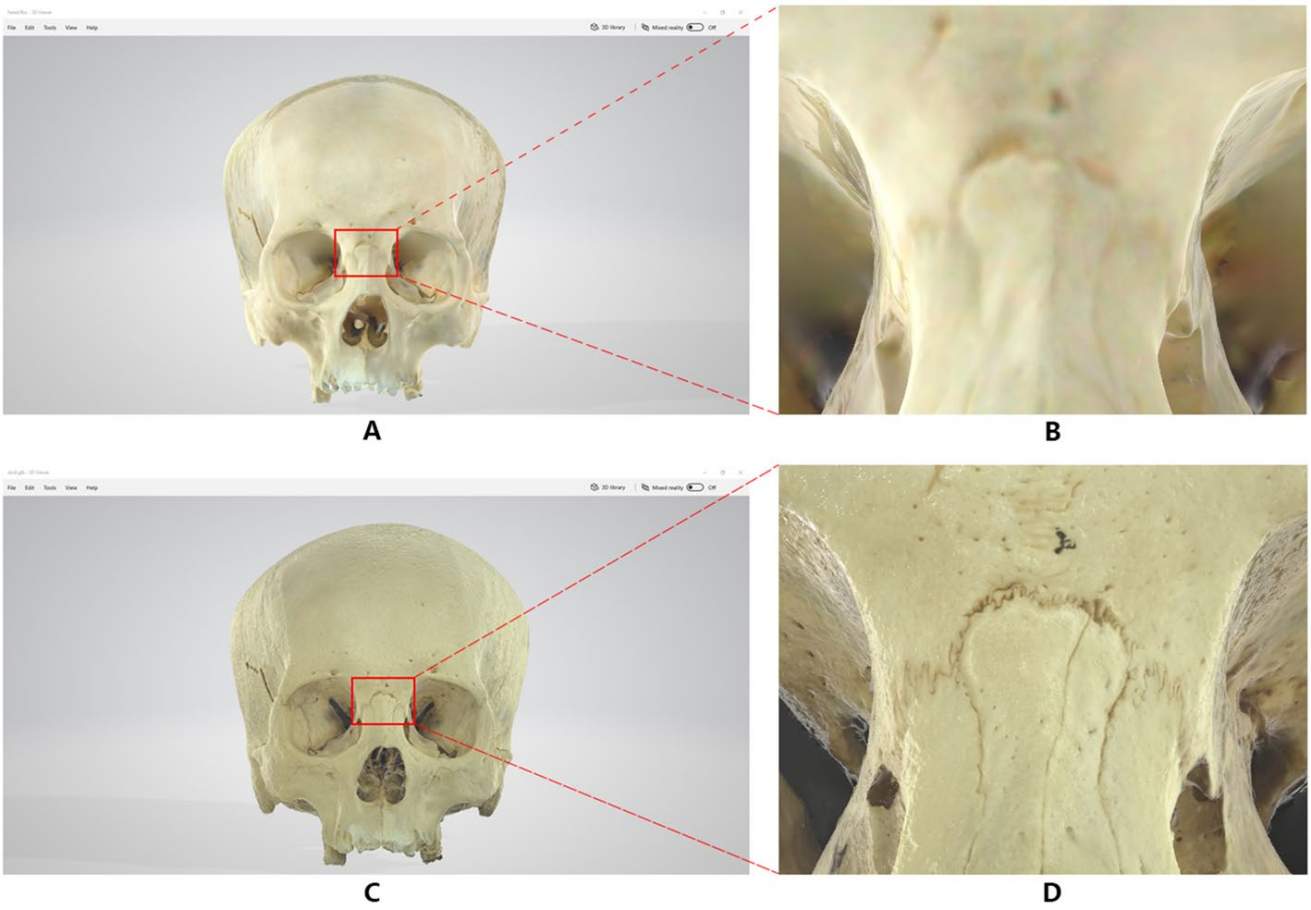
Comparison of two methods of skull imaging model data(1)

**Fig. 5:**
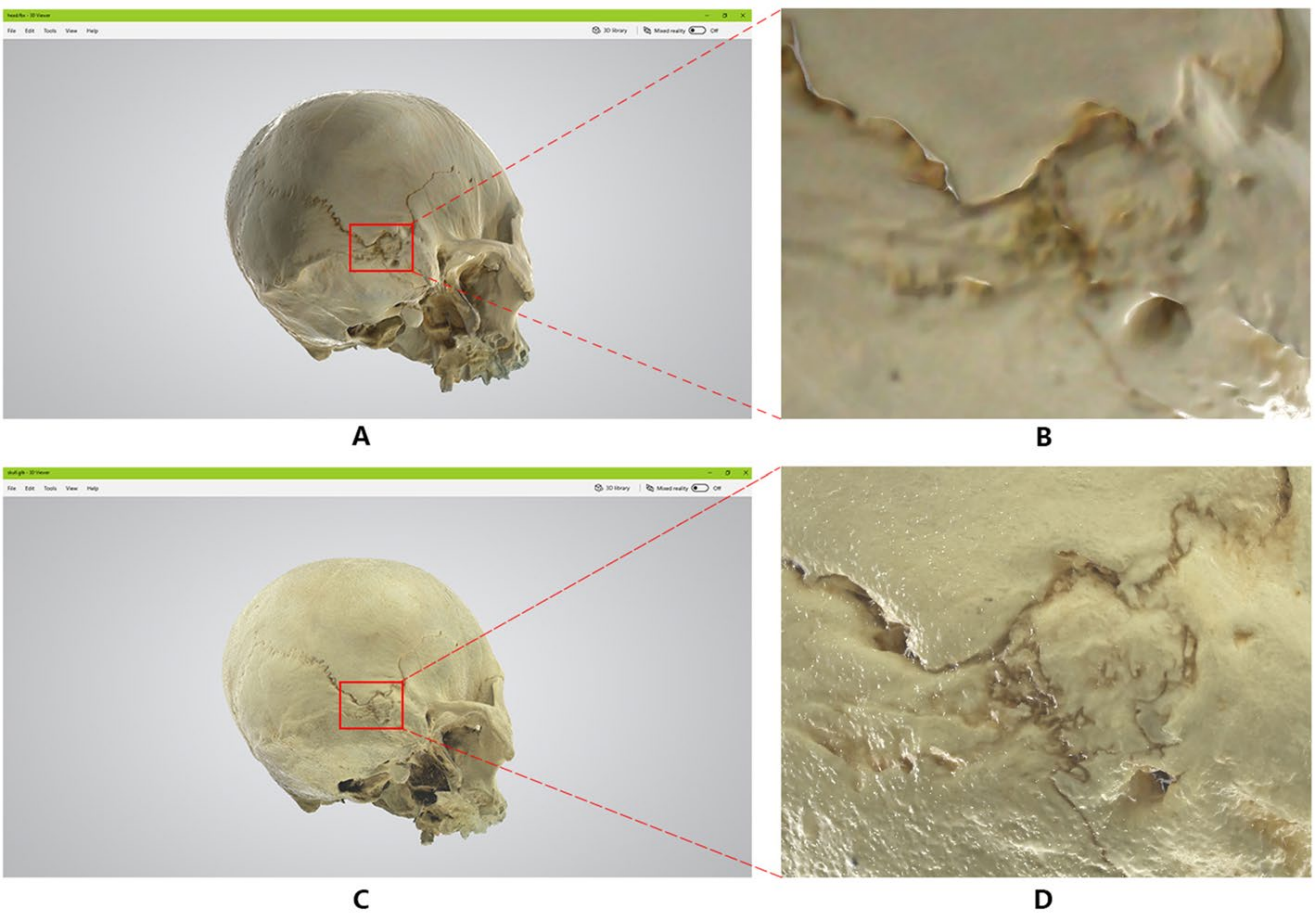
Comparison of two methods of skull imaging model data(2)

Use the 3D Viewer in Windows 10 to open the model GLB files generated by the two methods respectively to browse, take screenshots of the same parts of the two models and enlarge the part by 8 times for detailed comparison.

The model scanned and imaged by the handheld 3D scanner has low accuracy, fuzzy texture, dim color, easy to form visual dislocation in structure, and serious lack of details (see Fig. 4A.4B.5A.5B). Two-dimensional fitting three-dimensional models have high accuracy, fine textures, natural luster, can truly restore the structure of the skull, rich in details, and have a sense of essence, which can show the original sense of reality of the specimen (see Figs. 4C, D, 5C, D)).

In Fig. 4D, the nasal frontal suture, nasal maxillary suture, and frontal-maxillary suture can be seen, and even the nasal bony holes are clearly visible. In Fig. 5D, the complex intersection of the herringbone seam, the occipital suture and the apical breast suture is also the true restoration of the specimen itself.

### Details of the same location of the three facial nerves (Fig. 6)

**Fig. 6:**
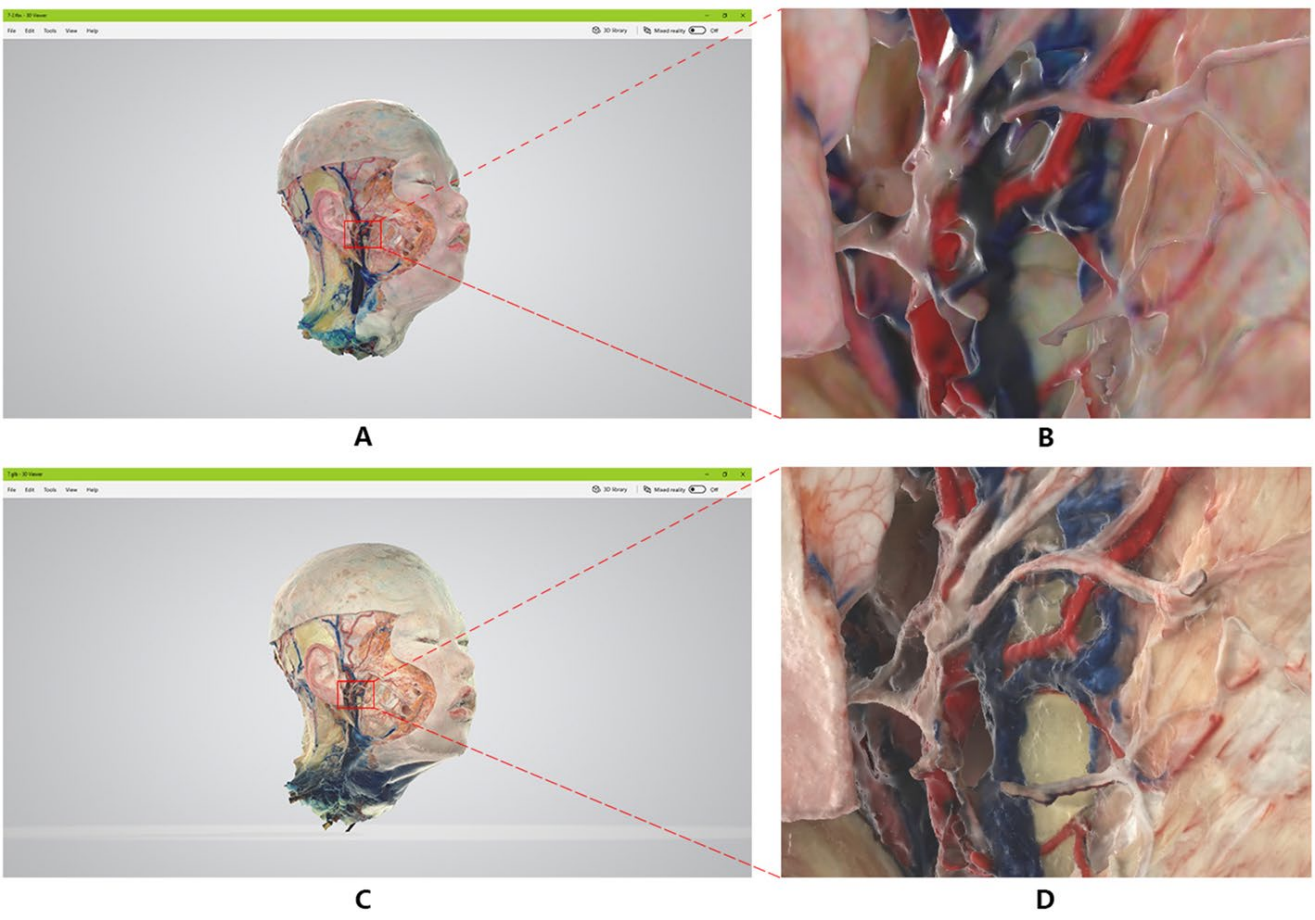
Comparison of two methods of facial nerves imaging model data

The image generation method is the same as result 2. From the local judgment and microscopic observation of the pictures obtained by the two sets of imaging methods, it can be seen that the edges of each tissue structure in the image generated by the handheld 3D scanner are not clear. Due to the low resolution generated by the equipment, the image trails are obvious, which leads to the collection of information The image generated at the missing place is broken or compensated by blunt calculations, and the authenticity of the specimen is lost (Fig. 6A.B).

The 2D fitting 3D reconstruction method produces a clear and complete image, and because of the high parameters of the image collected in the early stage of modeling, it has a higher resolution for different density tissues and complex shapes, so that the quality of the generated model is smoother. It is delicate, with clearer edge contours, less noise, and can truly restore different tissues such as facial nerve, arteriovenous, muscle, ear cartilage and parotid gland (Fig. 6C.D).

### Skull and facial nerves 3D video (video 1- 4)

3D model reading steps: Extract the file *.zip.Double click the model file *.glb.With Windows 10 software 3D viewer, click load settings in the environment & Lighting tab of 3D viewer, and select files in the pop-up dialog box settings.json.Click the grid & views tab, click projection, and select the orthographic mode.Press F9 on the keyboard to complete the environment setting.If you view another model again, double-click the model file without resetting the environment.When browsing use Windows 10's screen recording function to record, showing the process of using 3D Viewer to dynamically observe the GLB model. It can be seen that the clarity of the model presented by 2D fitting 3D and the true restoration of the specimen are significantly better than the hand-held scanner. (Video1 is 2D fitting 3D skull 3D viewer, Video2 is hand-held scanner skull 3D viewer, Video3 is 2D fitting 3D facial nerves 3D viewer, Video4 is hand-held scanner facial nerves 3D viewer).

### Comparative analysis of general data of three groups of students

There was no significant difference in gender and age among the three groups (*P* > 0.05) (Table 1).

**Table 1:** General data analysis

		traditional teaching group (*n* = 20)	hand held scanner 3D imaging group(*n* = 20)	2D fitting 3D method group(*n* = 20)	^2^/F	P
Gender	M(%)	14(70.0)	11(55.0)	12(60.0)	0.987	0.610
	F(%)	6(30.0)	9(45.0)	8(40.0)		
Age		20.6 ± 0.8	20.6 ± 0.7	20.3 ± 0.8	1.075	0.348

### Objective evaluation of three groups of students

The comparison of test results, clinical practice assessment and teaching satisfaction of the three groups shows that the performance of hand held scanner 3D imaging group is better than that of traditional teaching group (*P* < 0.01), and that of 2D fitting 3D method group is significantly better than that of traditional teaching group (*P* < 0.01)(Table 2).

**Table 2:** objective evaluation

	traditional teaching group (*n* = 20)	hand held scanner 3D imaging group(*n* = 20)	2D fitting 3D method group(n = 20)	X^2^/F	P
Test results	68.5 ± 4.9	76.6 ± 5.2	85.5 ± 5.3	55.073	< 0.001
Clinical practice assessment	66.6 ± 6.3	75.1 ± 5.5	82.6 ± 3.7	45.849	< 0.001
Teaching satisfaction	69.0 ± 4.2	72.0 ± 3.8	87.2 ± 4.1	118.191	< 0.001

### Subjective questionnaire analysis of three groups of students

After the teaching of the three groups, subjective questionnaires were conducted, which were ①Willing to continue this teaching mode, ②The ability to transform neurology from 2 to 3D, ③To deep the memory of the textbook, ④Intuitive understanding of Neurology, and ⑤Inter in the teaching method. (Table 3).

**Table 3:**
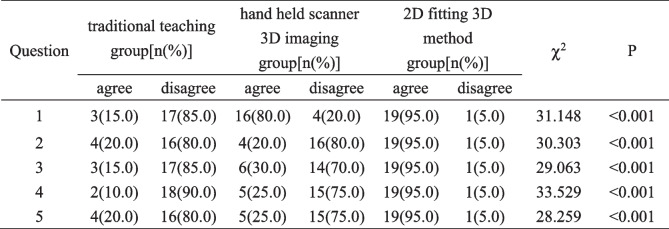
Subjective questionnaire analysis

## Discussion

As a course of morphology, observation of human specimen is the most direct method in anatomy teaching. The production of human body specimen needs to maximize the value of real specimen and demonstrate the human body structure in an all-round way, such that the students can truly understand and master the field. However, due to the shortage of human specimens, the requirement for every student cannot be satisfied. Usually, the students discuss and communicate after observing a specimen. Also, the lack of protection leads to the incompleteness of the specimen after repeated use. Most human specimens are soaked in formalin solution, leaving a pungent smell in the laboratory. If the ventilation device is not good, it will easily affect the students’ body, and the students’ interest in specimen observation is not high [5].

Neurosurgeons need microanatomics to be familiar with the brain details and construct 3D space imagination. The anatomists can achieve the purpose of learning but will spend plenty of time and energy. The pictures of the anatomical process are often presented in the form of 2D atlas. It is difficult for those who are not involved in the process of anatomy to form a 3D structure and understand the adjacent picture clearly. Thus, it has puzzled neurosurgery for many years.Three-dimensional (3D) modalities have gradually begun to supplement traditional 2-dimensionanl representations of dissections and illustrations [6].

At present, the practical application of virtual reality technology can vividly reproduce the process of human structural anatomical study. Students do not need to come in contact with the specimens and can still have an intuitive understanding of the anatomical process from different angles through virtual reality technology [7, 8]. However, due to the current limited hardware equipment of 3D imaging, especially for neural anatomy, it is difficult to restore the details of specimens truly. The method used in this study showed that the suture and foramen on the skull were clearly visible, and the terminal branches in the facial nerve anatomy were visible. Furthermore, no difference was detected between the naked eye and microscope results, and it was identical to that of the real cadaver head. Thus, each specimen was preserved permanently. However, the 3D images obtained by hand-held scanning are not sufficiently clear due to the small number of 3D model faces, insufficient model details, poor map resolution, and fuzzy map, resulting in the poor definition of the generated 3D image, which is different from the actual situation, and hence, it is difficult to meet the requirements of the specialty.

At present, the virtual simulation system of human anatomy is as follows: first, the medical imaging data are visualized, which is divided into volume rendering and surface rendering. Surface rendering uses segmentation technology to recognize and extract the contour of a series of 2D images, and finally, restore the 3D model of the detected object and displayed it in the form of a surface. Volume rendering is directly drawn on the original drawing, and the content requirement is smaller than that of the surface rendering. It takes a longer time than face rendering to evaluate the color and transparency of all pixels when switching a view angle. However, the content of the imaging display completely depends on the information provided by the imaging data. Interestingly, different types of examination, examination sequences, and examination parameters provide different tissue contrast; however, it is difficult to display all the tissue contrast of the human body in one examination sequence. Most human tissues can be restored to reality but need to be imaged according to a specific image sequence. It is difficult to deduce density differences due to the lack of tissue contrast in muscles, nerves, and other structures. Simultaneously, the generated image is displayed by computer simulation of light projection calculation, which lacks the reality of real natural light [9]. The second method is to present the human body and organs like animation 3D modeling, which is currently the most used technical method. Although the interactive operations, such as picking, rotating, hiding, and restoring objects, are performed, the lack of information sources and the realism of the images presented are unavoidable [10]. The third method is to form the point cloud image of the specimen through the 3D scanner and then input this high-definition image in the software. This process could achieve true restoration and reproduction, but it has the disadvantage that even the ultra-high-precision scanner cannot meet the accuracy requirements for anatomical reduction, especially the display of nerve fibers. Moreover, high-definition mapping in the later stage of ultra-high-precision scanning is complex, which requires significant efforts of the professionals. In addition, the performance requirements of the processing equipment are extremely high, which is extremely expensive [11].Many researchers are exploring a new method of 3D neuroanatomy and have made some progress [12].

The method used in this study can achieve real reduction. Compared to hand-held scanning, this method is more cost-effective than the cost of the equipment and the results. Moreover, the post-processing is easy to master, and the autopsy can be performed easily after learning, negating the need to seek professional help.

In this study, we reconstructed the 3D anatomical model and used the method of 2D photography. By calculating the relative spatial position of the 2D image, we constructed the 3D model. However, the specimen cannot be suspended and can only be placed on the working plane to achieve a horizontal 360° surrounding the model; moreover, this model has no bottom. Nonetheless, we require a complete model of 360°, which is a major limitation of this study. After repeated experiments, we applied the method of shooting the specimen in the horizontal direction first, then the vertical direction, and then adding the marker points in the software to realize the omnidirectional and complete 3D model. In order to facilitate the use, the 3D viewer software of Windows 10 was used to generate the image, which was saved as a GLB format file, and the file volume compressed further. It has the characteristics of small file volume and fast viewing by a 3D viewer.

## Supplementary Information

**Additional file 1.** 2D fitting 3D skull.

**Additional file 2.** Hand-held scanner skull.

**Additional file 3.** 2D fitting facial nerves.

**Additional file 4.** Hand-held scanner facial nerves.

## Data Availability

The datasets generated and/or analysed during the current study are not publicly available as they contain potentially identifable participant information. They are available from the corresponding author on reasonable request.
